# Hybrid Deep Neural Network Framework Combining Skeleton and Gait Features for Pathological Gait Recognition

**DOI:** 10.3390/bioengineering10101133

**Published:** 2023-09-27

**Authors:** Kooksung Jun, Keunhan Lee, Sanghyub Lee, Hwanho Lee, Mun Sang Kim

**Affiliations:** 1 Robocare, Seongnam 13449, Republic of Korea; ks_jun@robocare.co.kr; 2 School of Integrated Technology, Gwangju Institute of Science and Technology, Gwangju 61005, Republic of Korea; sang-hyub@gist.ac.kr; 3 Department of Otolaryngology-Head and Neck Surgery, Kosin University College of Medicine, Busan 49267, Republic of Korea; aya051@naver.com

**Keywords:** hybrid deep neural network, feature fusion, pathological gait recognition, skeleton-based gait analysis

## Abstract

Human skeleton data obtained using a depth camera have been used for pathological gait recognition to support doctor or physician diagnosis decisions. Most studies for skeleton-based pathological gait recognition have used either raw skeleton sequences directly or gait features, such as gait parameters and joint angles, extracted from raw skeleton sequences. We hypothesize that using skeleton, joint angles, and gait parameters together can improve recognition performance. This study aims to develop a deep neural network model that effectively combines different types of input data. We propose a hybrid deep neural network framework composed of a graph convolutional network, recurrent neural network, and artificial neural network to effectively encode skeleton sequences, joint angle sequences, and gait parameters, respectively. The features extracted from three different input data types are fused and fed into the final classification layer. We evaluate the proposed model on two different skeleton datasets (a simulated pathological gait dataset and a vestibular disorder gait dataset) that were collected using an Azure Kinect. The proposed model, with multiple types of input, improved the pathological gait recognition performance compared to single input models on both datasets. Furthermore, it achieved the best performance among the state-of-the-art models for skeleton-based action recognition.

## 1. Introduction

Gait represents crucial bioinformation, necessitating the proper integration of sensory, motor, and cognitive functions, and has consequently been the subject of extensive investigation for a considerable duration [1,2]. If the weakness of some body parts has a negative influence on those functions, the gait pattern can become abnormal and unbalanced. In other words, abnormal and unbalanced gait patterns indicate disorders of some body functions, and it is possible to find them by analyzing gait. Parkinson’s disease is a prominent example of a condition that manifests abnormal gait patterns, with numerous studies conducted on its gait characteristics [3,4,5,6]. It is marked by symptoms including a gradual reduction in walking speed, diminished swinging motion of the arms, shorter stride length, impaired balance, and a decline in the coordination of arm and trunk movements during walking. Additionally, numerous studies have explored the relationship between gait patterns and other specific diseases, including autism spectrum disorder [7,8], stroke [9,10], Alzheimer’s disease [11], vestibular problems [12,13,14], and functional gait disorders [15,16]. Furthermore, gait patterns have been used in practice to support doctor or physician decisions for patients. There are many research groups studying gait patterns and various research results continue to be published.

Recognizing pathological gait patterns helps to diagnose a disease and even to find a presymptom of a disease before it worsens. Therefore, there have been many approaches for pathological gait recognition using various sensors, such as inertial measurement units (IMUs), plantar foot pressure sensors, motion capture systems, and depth cameras. Sensor-based systems have many advantages. They make it possible to automatically prescreen for specific diseases without visiting a hospital. People hardly realize whether their gait patterns are changed or not because they change gradually. It might be too late if they realize their abnormal gait by themselves. On the other hand, sensor systems can analyze a gait pattern with objective standards, so it is possible to detect abnormal gaits in the early phase of a disease, and patients can receive proper treatment before the disease worsens. Therefore, if a sensor-based pathological gait recognition system is installed in a home or elderly care center and people conduct gait analysis periodically, specific diseases can be prescreened without visiting a hospital.

A depth camera was used to recognize pathological gaits in this study. A depth camera, such as Kinect (Microsoft Corp., Redmond, WA, USA), Astra (Orbbec 3D Technology International, Inc., Troy, MI, USA), and Realsense (Intel Corp., Santa Clara, CA, USA), can obtain not only RGB data but also depth data for each pixel. The collected RGB and depth data can be used to simulate the human skeleton, which contains three-dimensional positional information of each joint. A depth camera can measure gait data without attaching sensors or markers, whereas an IMU and motion capture system require the attachment of sensors or markers, which can make the walker feel uncomfortable and walk unnaturally. Furthermore, a depth camera can obtain information on all body joints. A depth camera-based gait analysis system is simple to operate and has a relatively low cost and reasonable accuracy, so it can be operated in various environments. Therefore, many studies have used human skeleton data obtained through a depth camera to recognize pathological gaits [3,4,11,17,18,19,20,21,22,23,24,25,26,27].

In the domain of skeleton-based pathological recognition, numerous research studies have been conducted utilizing machine learning algorithms. For instance, Li et al. [3] proposed a method to classify normal individuals and patients with hemiplegia and Parkinson’s disease using k-nearest neighbors. They used a covariance matrix representing joint motions and speeds extracted from the skeleton sequence. Dranca et al. [4] introduced a machine learning-based method to classify Parkinson’s disease stages. They extracted features from the skeleton by applying correlation-based feature selection, information gain, or consistency subset evaluation. Seifallahi et al. [11] proposed a method to detect Alzheimer’s disease by employing a support vector machine (SVM) classifier with a Gaussian kernel. Gait parameters, such as time walking, step length, step number, stride length, gait cycle, and stride velocity, were fed to the classification model in their work. Bei et al. [22] proposed a method to detect movement disorders using machine learning algorithms. Gait parameters, such as gait symmetry, step length, and gait cycle, were fed to the classification model in their study. Chakraborty et al. [23] introduced a method for the automatic diagnosis of cerebral palsy gait using a multi-Kinect system and SVM-based classification model. They extracted spatiotemporal features from the skeleton and used them as the input data to the classifier. Chakraborty et al. [24] employed a multiple adaptive regression splines model to recognize equinus foot deformity gait. The hip, knee, and ankle angles of both sides were extracted from the skeleton and used as the input data in their work.

Deep neural network models have also been applied to skeleton-based pathological gait recognition. For instance, Guo et al. [17] proposed a bidirectional long short-term memory (LSTM)-based model to classify normal, in-toeing, out-toeing, drop-foot, pronation, and supination gaits. The lower limb skeleton was used to extract statistical features and angle sequences in their study. Tian et al. [25] proposed a spatiotemporal attention-enhanced gait-structural graph convolutional network (AGS-GCN) to recognize abnormal gaits. They used the lower limb joints and spine base to extract spatiotemporal gait parameters, such as the joint trajectory, joint angle, and gait link. Sadeghzadehyazdi et al. [26] proposed a hybrid model composed of a convolutional neural network (CNN) and LSTM to model spatiotemporal patterns for gait anomaly recognition. They used normalized joints for the classification. Kim et al. [27] applied a spatiotemporal GCN with an attention mechanism to the spatiotemporal features extracted from skeleton data for the recognition of abnormal gaits.

Most existing methods for skeleton-based pathological gait recognition use gait features extracted from raw skeletons, such as static gait parameters [11,22,23] and joint angle sequences [4,17,24]. They have shown their effectiveness in recognizing pathological gaits. Gait features effectively represent gait abnormalities, so they can be interpreted more easily than raw skeleton data. However, this does not mean that they can represent all the important information of the raw skeleton data. On the other hand, the raw skeleton data include all the important information; however, it is difficult to understand a gait abnormality because of the complicated structure and large data size. Recently, studies inputting the skeleton itself into a model have been published [28,29,30]. They showed the possibility of recognizing pathological gaits by interpreting the skeleton data, but gait features were not considered at all. Prior studies have not extensively explored a hybrid approach that combines the advantages of both gait features and raw skeleton data. This means that while gait features provide valuable insights into gait abnormalities, they may not fully exploit the richness of information contained in the raw skeleton data. Thus, there is an opportunity to investigate novel methods that leverage both gait features and raw skeleton data to enhance the recognition of pathological gaits. Following this observation, our motivation evolved into exploring novel methods that harness the complementary strengths of both gait features and raw skeleton data to improve pathological gait recognition.

We hypothesized that using both gait features and raw skeleton data could further improve the performance of pathological gait recognition since they have different advantages and representations. Gait parameters are the most compressed form to effectively represent the abnormality of a gait, joint sequences are focused on showing the bending and balancing abilities of lower limb joints, and raw skeleton sequences preserve all important information for pathological gait recognition and show the overall movement of the whole body during walking. Using these together can facilitate a model to converge to the global minima since gait abnormalities can be interpreted through a variety of perspectives. However, a method to use all of them together for pathological gait recognition has not yet been proposed. In consideration of these factors, we have innovatively introduced a novel methodology encompassing the concurrent utilization of gait parameters, joint sequences, and raw skeleton sequences, marking a pioneering advancement in the field.

In this paper, we propose a novel hybrid deep learning model designed to maximize the utilization of raw skeleton data and gait features for pathological gait recognition. Since the input data have different characteristics, we applied different deep learning architectures to encode each input data effectively. A graph convolutional network (GCN), recurrent neural network (RNN), and artificial neural network (ANN) are used to encode the raw skeleton sequences, the joint angle sequences, and the gait parameters, respectively. Their outputs are fused together and fed into the final classification layer. This fusion of features can be achieved through concatenation, with further performance enhancements achievable through feature selection or weighting techniques. This involves the selection or assignment of weights to features and matching scores that demonstrate low correlation, as exemplified by [31], and high discrimination, as illustrated by [32].

The primary objective of this study is to demonstrate improved performance in recognizing pathological gait patterns through the fusion of raw skeleton data and gait features with our hybrid deep learning model designed to synergize diverse input types. Given the inherent diversity in the characteristics of input data, our approach incorporates distinct deep learning architectures tailored for encoding each specific data type. Our proposed model stands as an innovative contribution by integrating raw skeleton sequences, joint angle sequences, and gait parameters, marking the first of its kind in the realm of pathological gait recognition. To substantiate the efficacy of this pioneering model, comprehensive evaluations were conducted on diverse pathological gait datasets, including a simulated pathological gait dataset and a vestibular disorder gait dataset, both meticulously collected utilizing Azure Kinect. Furthermore, rigorous comparative analyses were conducted against state-of-the-art models specialized in skeleton-based action recognition.

## 2. Materials and Methods

**Figure 1:**
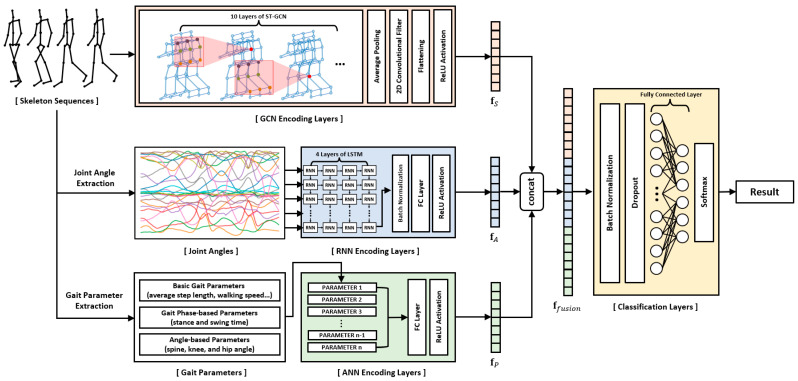
Structure of the proposed multi-input hybrid deep neural network. The skeleton sequences, joint angles, and gait parameters are input to the GCN, RNN, and ANN layers, respectively. Each encoding layer encodes the input data into a one-dimensional feature vector. The outputs of each encoding layer are concatenated together and fed to the final classification layer.

Most studies in the field of skeleton-based pathological gait recognition have traditionally focused on two primary approaches: utilizing raw skeleton sequences directly or extracting gait features, such as gait parameters and joint angles, from these raw sequences. However, these studies have often treated each data type in isolation or separately. In this study, we aim to address the potential for improved recognition performance by effectively combining different types of input data. To achieve this, we propose a novel deep neural network model. This model adopts a hybrid deep neural network framework, consisting of GCN, RNN, and ANN layers. Each of these components is specifically designed to encode skeleton sequences, joint angle sequences, and gait parameters, respectively. The extracted features from these three distinct data types are then fused together and input into the final classification layer. A comprehensive illustration of this network is presented in Figure 1. By employing this innovative approach, our research aims to enhance pathological gait recognition performance. To demonstrate the effectiveness of our proposed model, we collected two skeleton datasets using Azure Kinect: a simulated pathological gait dataset and a vestibular disorder gait dataset, and subsequently conducted evaluations using these datasets.

### 2.1. Data Acquisition

A depth camera-based skeleton data collection system was developed in the healthcare robotics laboratory at the Gwangju Institute of Science and Technology, Korea. An Azure Kinect and the corresponding body tracking software development kit (SDK) developed by Microsoft were used to collect the skeleton data. The system collected the data while a subject walked straight forward toward the sensor approximately 4 m away. The sensor was calibrated by recognizing an ArUco marker [33] to collect the data in the same coordinate system. The XYZ coordinate system of the sensor was transformed to the XYZ coordinate system of the marker. The 3-dimensional position of each vertex of the marker was measured using the sensor, and the transformation matrix was obtained. Each data example contained 80–120 frames of skeleton data with an average collection rate of 22.7 fps. We evaluated the proposed hybrid model on two skeleton datasets (a simulated pathological gait dataset and a vestibular disorder gait dataset) collected by the data acquisition system. The evaluation encompassed the assessment of the model’s proficiency in handling intricate gait classification tasks, specifically its ability to differentiate among six distinct gait patterns within the simulated pathological gait dataset. Simultaneously, the vestibular disorder gait dataset, comprising genuine patient data, facilitated an examination of the model’s practicality and suitability in real world contexts, particularly when confronted with datasets originating from individuals afflicted by vestibular disorders. This comprehensive evaluation allowed us to assess both the model’s technical capabilities and its real-world applicability.

#### 2.1.1. Simulated Pathological Gait Dataset

**Figure 2:**
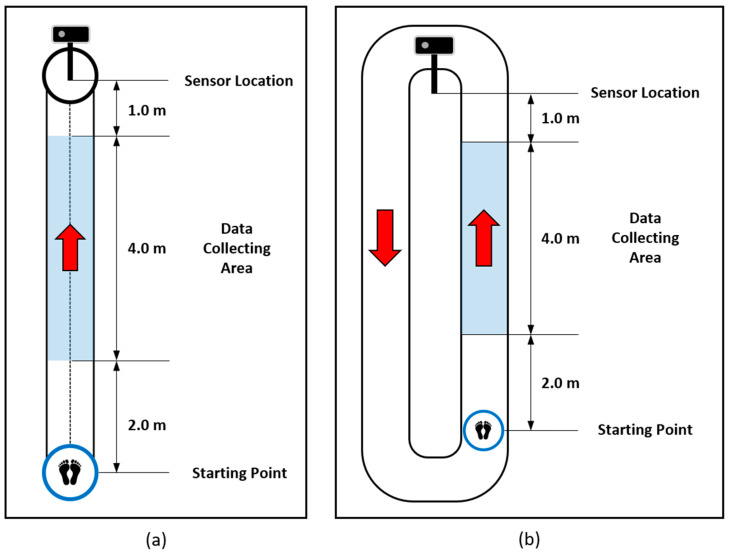
Data acquisition environment: (**a**) simulated pathological gait dataset and (**b**) vestibular disorder gait dataset.

Most previous studies conducted binary classification by differentiating pathological gaits from a healthy gait [11,22,23,24,25], and there have also been a few studies recognizing various and complicated pathological gaits [3,17,27]. Multilabel pathological gait classification is much more difficult than binary classification and can help to evaluate a model from various directions. Therefore, we collected various and complicated pathological gait data. Normal gait and five pathological gaits, i.e., antalgic, steppage, lurching, stiff-legged, and Trendelenburg gaits, were collected through simulations of 12 healthy subjects. They were asked to walk along a 7 m walkway, as shown in Figure 2a. The characteristics and causes of each pathological gait are described in detail in [20]. The subjects understood the mechanical reason for the pathological gaits before data collection, so they could simulate the pathological gaits similarly to real patients. The data collection was conducted under strict expert supervision. The subjects were asked to walk with each gait 20 times. Therefore, 1,440 examples (12 subjects × 6 gaits × 20 walks) were included in this dataset. Furthermore, we augmented the dataset by reversing the left and right sides of the skeleton, so 2,880 examples were used for the experiments. Through this dataset, the performance of the proposed model on the classification of complicated pathological gaits was evaluated.

#### 2.1.2. Vestibular Disorder Gait Dataset

Regardless of how well the simulated gait data were classified, it was difficult to verify their practicality in the real world. Therefore, we collected real patient data to evaluate the practical applicability of the proposed model. Gait data of real patients with vestibular problems were obtained with the support of the Kosin University Gospel Hospital. The subjects were asked to walk two laps around a 16 m track. We collected the skeleton data while the subjects were walking on the data collecting area, as shown in Figure 2b, because the sensor and the body tracking SDK do not guarantee high-quality skeleton data when the human is not facing the sensor. Thirty-three healthy subjects (12 females and 21 males, with a mean age of 38.9 ± 16.4 years) and 128 patients with a vestibular disorder (94 females and 34 males, with a mean age of 58.5 ± 13.5 years) participated in the data collection. Since there was a large difference in the average age between the healthy subjects and the patients, we downsampled the patient data and made a balanced group with 33 healthy subjects and 54 patients with a vestibular disorder (34 females and 20 males, with a mean age of 46.1 ± 10.4 years). Ten data examples whose sequences were less than 90 were excluded from the evaluation. Therefore, we evaluated the proposed model on the all-subject group with 312 data examples (161 subjects × 2 walks—10 exemptions) and the balanced group with 170 data examples (87 subjects × 2 walks—4 exemptions).

### 2.2. Graph Convolutional Network for Skeleton Data

CNNs are renowned for their effectiveness in tasks involving visual data analysis, primarily due to their ability to capture intricate spatial relationships between pixels within an image, a feature that sets them apart [34]. In contrast, a GCN is particularly well suited for tasks such as node classification and link prediction within data structured as graphs [35]. Examples of such data encompass social networks, chemical molecules, and skeletal datasets. A GCN is known as the most powerful structure for skeleton-based action recognition. Yan et al. [36] first introduced a method to apply a spatial–temporal graph convolutional network (ST-GCN) for skeleton-based action recognition. They suggested a way to efficiently process skeleton sequences by simultaneously understanding the spatial and temporal characteristics of the skeleton data. Subsequently, many modified GCN structures have been introduced, and the performance of skeleton-based action recognition continues to improve [37,38,39,40,41,42,43]. In this study, we adopt the ideas and formulations of the ST-GCN proposed in [36] to encode skeleton data.

The skeleton sequences are denoted as a spatial–temporal graph G=(V,E). The node set $V=\{\left.v_{ti}\right|t=1,...,T,i=1,...,N\}$ contains all the joints in the skeleton sequences, where T and N denote the number of sequences and the number of joints, respectively. Every node includes three channels $v_{ti}=(x_{ti},y_{ti},z_{ti})$ since we use the 3-dimensional position information of each joint. The edge set E is divided into two subsets, the edge set of naturally connected human joints (intraskeleton edges) and the edge set of consecutive frames on the same joint (interframe edges), which are denoted by $E_{S}=\{v_{ti}v_{tj}|(i,j)\in H\}$ and $E_{F}=\{v_{ti}v_{(t+1)i}\}$, respectively, where H is the set of naturally connected joints.

The spatial convolution operation for a joint node $v_{ti}$ can be formulated as the following equation:

(1) $$F_{out}(v_{ti})=\sum_{v_{tj}\in B(v_{ti})}\frac{1}{Z_{ti}(v_{tj})}F_{in}(p(v_{ti},v_{tj}))\cdot w(M_{ti}(v_{tj}))$$

where $F_{out}$ and $F_{in}$ denote the output and input features of the GCN, respectively. $Z_{ti}(v_{tj})$ denotes a normalization term to balance the contribution of each subset. A sampling function $p(v_{ti},v_{tj})$ is defined on the neighbor set $B(v_{ti})=\{v_{tj}|d(v_{tj},v_{ti})\leq D\}$, where $d(v_{tj},v_{ti})$ denotes the minimum length of the path from $v_{tj}$ to $v_{ti}$. A weight function w is defined by partitioning the neighbor set into subsets with a numeric label based on a mapping function $M_{ti}(v_{tj})$ that maps the neighbor nodes into their subset labels.

Yan et al. [36] extended the concept of a neighborhood to cover temporally consecutive joints by modifying the neighbor set $B(v_{ti})$ and defining a spatial–temporal mapping function $M_{ST}$ as follows:

(2) $$B(v_{ti})=\left\{\left.v_{qj}\right|d(v_{tj},v_{ti})\leq K,\left|q-t\right|\leq \left\lfloor \Gamma /2\right\rfloor \right\}$$

(3) $$M_{ST}(v_{qj})=M_{ti}(v_{tj})+\left(q-t+\left\lfloor \Gamma /2\right\rfloor \right)\times K$$

where K and Γ denote the number of subsets derived by mapping function $M_{ti}(v_{tj})$ and the range of interest for temporal convolution, respectively.

In this study, we implemented a multilayer ST-GCN to encode the skeleton sequences into a 1-dimensional feature vector $f_{S}$. Global pooling was applied to the outputs of the ST-GCN layers, and then a convolutional operation was conducted to extract the feature vector with a specific size. Finally, the multichannel output was resized to a 1-dimensional vector.

### 2.3. Recurrent Neural Network for Joint Angles

**Figure 3:**
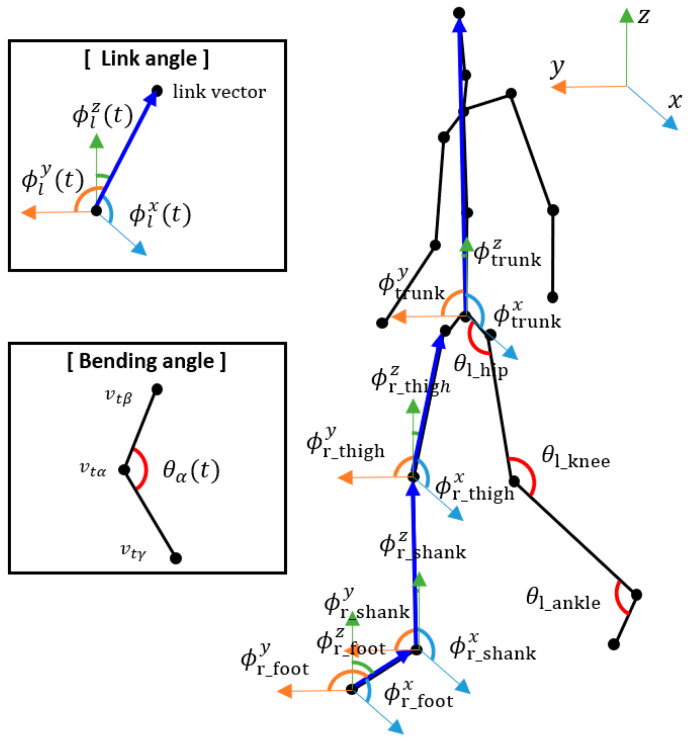
A sample of the joint angles. The joint angles include the link angles and the bending angles.

We extracted the joint angle sequences from the skeleton sequences and used them as another input to the proposed hybrid model. We extracted the bending angles and link angles of specific joints according to [17], which showed their effectiveness on pathological gait recognition. Examples of the joint angles are shown in Figure 3.

The bending angle of joint α∈{left_hip, right_hip, left_knee, right_knee, left_ankle, right_ankle} at time t was calculated according to the following equation:

(4) $$\theta_{\alpha }(t)=\cos^{-1}\left(\frac{(v_{t\beta }-v_{t\alpha }{)}^{2}+(v_{t\gamma }-v_{t\alpha }{)}^{2}-(v_{t\gamma }-v_{t\beta }{)}^{2}}{2(v_{t\beta }-v_{t\alpha })\cdot (v_{t\gamma }-v_{t\alpha })}\right)$$

where β and γ denote the joints connected to joint α. For example, $\theta_{left\_knee}(t)$ was calculated using the left knee, left hip, and left ankle joints.

The link angle of link l∈{left_thigh, right_thigh, left_shank, right_shank, left_foot, right_foot, trunk} about x-axis at time t was calculated using the following equation:

(5) $$\phi_{l}^{x}(t)=\cos^{-1}\left(\frac{(v_{tm}-v_{tn})\cdot u_{x}}{\left|v_{tm}-v_{tn}\right|\left|u_{x}\right|}\right)$$

where m and n denote the joints used to construct link l. The thigh consisted of the knee and hip joints, the shank consisted of the ankle and knee joints, the foot consisted of the tiptoe and ankle joints, and the trunk consisted of the pelvis and head joints. $u_{x}$ denotes the unit vector along x-axis. $\phi_{l}^{y}(t)$ and $\phi_{l}^{z}(t)$ were calculated similarly by utilizing $u_{y}$ and $u_{z}$ instead of $u_{x}$.

A total of 25 angles were extracted from each skeleton and the sequences of the angles were fed to the RNN encoding layers. An RNN is a specialized architecture for handling sequential data, including time series data like stock prices, audio data, and skeletal sequences. Since the structure of a basic RNN has a long-term dependency problem in which the influence of the previous information continues to decrease as the hidden state is updated for long-term sequential data, we adopt LSTM to construct the RNN layers. LSTM can solve the problem by employing a gated structure to update the hidden state $h_{t}$. The variables for the gated structure, i.e., the forget gate $f_{t}$, input gate $i_{t}$, output gate $o_{t}$, and cell state $C_{t}$, are formulated as follows:

(6) $$f_{t}=\sigma \left(W_{xf}x_{t}+W_{hf}h_{t-1}+b_{f}\right)$$

(7) $$i_{t}=\sigma \left(W_{xi}x_{t}+W_{hi}h_{t-1}+b_{i}\right)$$

(8) $$o_{t}=\sigma \left(W_{xo}x_{t}+W_{ho}h_{t-1}+b_{o}\right)$$

(9) $$C_{t}=f_{t}\circ C_{t-1}+i_{t}\circ \tan h\left(W_{xC}x_{t}+W_{hC}h_{t-1}+b_{C}\right)$$

where x, W, b, σ, and ∘ denote the input, weights, biases, sigmoid function, and elementwise product, respectively. The hidden state $h_{t}$ and the output $y_{t}$ can be updated as follows:

(10) $$h_{t}=o_{t}\circ \tan h\left(C_{t}\right)$$

(11) $$y_{t}=W_{y}\cdot h_{t}+b_{y}.$$

We constructed a multilayer LSTM to encode the joint angle sequences into a 1-dimensional feature vector $f_{A}$. The output of the multilayer LSTM operation was the last hidden state of the final LSTM layer, which was fed to the fully connected layer to extract a feature vector with a specific size as follows:

(12) $$f_{A}=\mathrm{ReLU}(W_{A}\mathrm{LSTM}(x_{A})+b_{A})$$

where $x_{A}$, $W_{A}$, $b_{A}$, and LSTM(⋅) denote the input joint angle sequences, weight, bias, and multilayer LSTM operation, respectively.

### 2.4. Artificial Neural Network for Gait Parameters

Gait parameters are important indicators to recognize pathological gaits [44,45,46]. We obtained basic gait parameters, gait phase-based parameters, and angle-based parameters using 3-dimensional skeleton sequences. These parameters encompass various aspects of walking and are instrumental in assessing an individual’s gait.

The basic gait parameters include average step length, step length asymmetry, step width, and walking speed. Average step length measures the typical distance a person covers with a single step, typically from one heel to the other throughout a complete walking cycle. Step length asymmetry highlights the difference in step lengths between the left and right legs during walking, providing insights into the symmetry and balance of steps. Step width assesses the lateral distance between the feet at their widest point during the gait cycle, indicating whether steps are wide or narrow. Walking speed represents the rate of forward movement during walking, offering information about walking pace.

The gait phase-based parameters encompass stance and swing time on both legs. Swing time on both legs indicates the duration when the leg is not in contact with the ground during the gait cycle, typically measured in seconds, covering the time from foot lift off to foot strike. Stance time on both legs measures the duration of the gait cycle when the leg is in contact with the ground, providing essential information about how long each leg supports the body’s weight during walking.

Lastly, the angle-based parameters encompass mean, minimum, and maximum values for frontal spine angle, lateral spine angle, knee angle, and hip angle. Frontal spine angle measures how the spine deviates from the vertical plane when viewed from the front, particularly relevant in posture and gait analysis for detecting deviations in the frontal plane. Lateral spine angle quantifies spine deviation from the vertical plane when viewed from the side, providing insights into body alignment during walking, especially lateral deviations. Knee angle on both legs describes the angle formed at the knee joint between the thigh and the lower leg for both legs during different phases of the gait cycle, offering insights into knee joint flexion and extension. Hip angle on both legs measures the angle at the hip joint between the thigh and the pelvis on both sides of the body during walking, reflecting hip movement and positioning throughout the gait cycle.

It is important to note that all parameters, except angle-related ones, fall within the range of 0 to 2, while angle-related parameters could potentially range from 0 to 180 degrees. To ensure consistency for input into an artificial neural network, these angle-related parameters were normalized by dividing them by 100. This normalization process brings them into the same 0 to 2 range as the other parameters, improving the training stability and convergence of an artificial neural network.

We used the extracted gait parameters as the final input data to the proposed model. A fully connected ANN was used to encode the gait parameters into a 1-dimensional feature vector $f_{P}$ with a specific size as follows:

(13) $$f_{P}=\mathrm{ReLU}(W_{P}x_{P}+b_{P})$$

where $x_{P}$, $W_{P}$, and $b_{P}$ denote the input gait parameters, weight, and bias, respectively.

### 2.5. Fusion of Features and Classification

The skeleton sequences, joint angle sequences, and gait parameters were input to the GCN-based, RNN-based, and ANN-based layers, respectively. The features extracted from each layer were concatenated into a 1-dimensional vector, and the integrated features were fed to the final classification layer using the following equations:

(14) $$f_{fusion}=\mathrm{concatenate}(f_{S},f_{A},f_{P})$$

(15) $$y=\mathrm{softmax}(W_{y}f_{fusion}+b_{y})$$

where y, $W_{y}$, and $b_{y}$ denote the output, weight, and bias of the fully connected layer to recognize the gait type. Batch normalization and dropout were conducted before the operation of the fully connected layer to prevent overfitting. The index of the maximum value in y is the recognized gait type.

A cross-entropy loss function was adopted to calculate the loss $L_{CE}$, and L2 regularizations were applied to avoid overfitting as follows:

(16) $$L_{CE}(y,\overset{̑}{y})=-\sum_{i=1}^{C}y_{i}\log({\overset{̑}{y}}_{i})+\frac{\lambda }{2}{\left|W\right|}^{2}$$

where λ and W are the regularization parameter and trainable weights, respectively. Table 1 provides an exhaustive delineation of the intricate configuration of the multi-input hybrid neural network.

### 2.6. Training Environment

The experimental configurations of the computer are an Intel^®^Core™ i7-7700K central processing unit, an NVIDIA GeForce RTX 2080 Ti graphics processing unit, and 64 GB of random access memory. In this study, PyTorch and scikit-learn were adopted to implement the deep learning and machine learning models, respectively. All deep learning models used in the experiments were trained under the same training options (a batch size of 50, 200 training epochs, early stopping, cross-entropy loss, and the Adam [47] optimizer). However, some training options, such as the learning rate and weight decay, were set according to the suggested training configurations of each state-of-the-art model.

## 3. Results

The performance of the proposed model for skeleton-based pathological gait recognition was evaluated on the simulated pathological gait dataset and the vestibular disorder gait dataset. They include different gait abnormalities and have different subject configurations, so it is meaningful to evaluate the proposed model using both datasets and to compare the results. Furthermore, diverse state-of-the-art models for skeleton-based action recognition, such as the ST-GCN [36], hierarchical cooccurrence network (HCN) [37], decoupling GCN [38], two-stream adaptive graph convolutional network (2s-AGCN) [39], multistream attention-enhanced adaptive graph convolutional network (MS-AAGCN) [40], part-based graph convolutional network (PB-GCN) [41], decoupled spatial–temporal attention network (DSTA-NET) [42], and channelwise topology refinement graph convolutional network (CTR-GCN) [43], were compared to demonstrate the effectiveness of the proposed hybrid model.

### 3.1. Evaluation on the Simulated Pathological Gait Dataset

Leave-one-subject-out cross validation was applied to the simulated pathological gait dataset to compensate for the small number of subjects. The number of skeleton and joint angle sequences used as the input data was set to 100. We abandoned the last 10 sequences and used the 100 sequences immediately preceding them because the skeleton data were noisy if the distance between the human and the depth camera was too close.

We compared the performances of various models when only a single type of input data was used, as shown in Table 2. Classic machine learning-based models, such as AdaBoost, a decision tree, Gaussian Naïve Bayes, random forest, k-nearest neighbor (k-NN), and SVM, and deep neural network-based models, such as a multilayer perceptron (MLP), GRU [20], LSTM [20], and ST-GCN [36], were used for the comparison. For the gait parameters, the MLP achieved the best performance with 90.49% accuracy, and the SVM showed the second highest accuracy with 88.47% accuracy. For the joint angles, the RNN architectures showed their powerfulness in analysis. LSTM [20] achieved the best performance with 93.30% accuracy, and the GRU [20] showed the second highest accuracy with 92.92% accuracy. For the skeleton data, the ST-GCN [36] achieved the best performance with 96.94% accuracy, and the GRU [20] showed the second highest accuracy with 95.83% accuracy. Among the three input data types, the highest accuracy was achieved when the skeleton data were input to the ST-GCN [36] model.

**Figure 4:**
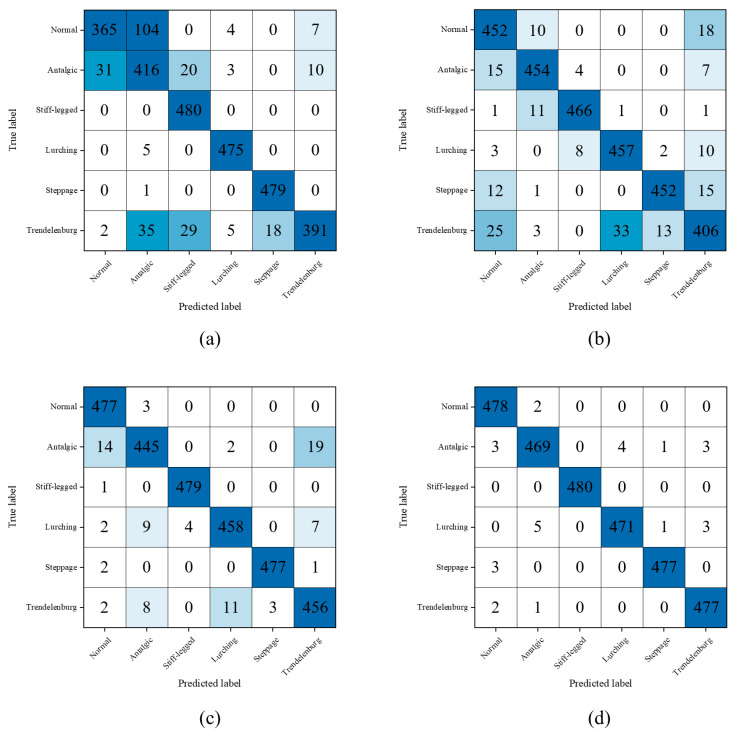
Confusion matrices of the best single input models and the proposed multi-input model on the simulated pathological gait dataset: (**a**) the gait parameters were input to the MLP model; (**b**) the joint angle sequences were input to the LSTM [20] model; (**c**) the skeleton sequences were input to the ST-GCN [36] model; and (**d**) all types of inputs were input to the proposed model.

The proposed hybrid model fed with multiple types of input data achieved 99.03% accuracy, which was higher than that of the best single input models for each data type. The fusion of the gait features and the raw skeleton sequences improved the performance of pathological gait classification. Figure 4 shows the confusion matrices of the results of the single input models and the proposed hybrid model. The parameter-based classification had poor performance in classifying normal and Trendelenburg gaits, which were misclassified 115 and 89 times, respectively. The joint angle-based classification showed better performance than the parameter-based classification for this dataset. However, it showed poor performance in classifying the Trendelenburg gait with 74 misclassifications. The skeleton-based classification showed the best performance among the single input models. The overall gaits were well classified, but the antalgic gait classification seemed to need further improvement. The proposed multi-input hybrid model achieved the best performance by classifying the normal and five pathological gaits with a few errors.

The proposed model showed the highest accuracy among the state-of-the-art models for skeleton-based action recognition, as shown in Table 3. The CTR-GCN [43] and 2s-AGCN [39] showed the second and the third most accurate performances with 98.75% and 98.06% accuracy, respectively. The GCN-based models showed better performance than the RNN-based models. Although the state-of-the-art GCN models showed their powerfulness in skeleton-based pathological gait recognition, they could not surpass the performance of the proposed hybrid model combining the skeleton data and gait features.

### 3.2. Evaluation on the Vestibular Disorder Gait Dataset

We evaluated the proposed model using a real patient dataset to verify its practical applicability to the real world. Fivefold cross validation was applied for the evaluation of the vestibular disorder gait dataset since it had a large number of subjects. The number of skeleton and joint angle sequences used as the input data was set to 80. As before, we abandoned the final 10 sequences and used the 80 sequences immediately preceding them.

The proposed model was compared with state-of-the-art models for skeleton-based action recognition. The statistical indices (accuracy, sensitivity, specificity, and precision) of the models were evaluated on the all-subject group and the balanced group. Table 4 shows the accuracy, sensitivity, specificity, and precision for the all-subject group with the true positive (TP), false positive (FP), true negative (TN), and false negative (FN) values. The proposed model achieved 91.03% accuracy, 93.15% sensitivity, 82.81% specificity, and 95.45% precision. The accuracy and sensitivity of the proposed model were the highest among the models. MS-AAGCN [40] and CTR-GCN [43] showed the second and third highest accuracies of 89.74% and 89.42%, respectively. DSTA-NET [42] achieved the second highest sensitivity of 91.94%. The specificity and precision of the proposed model were not the highest among the models. The MS-AAGCN [40] and CTR-GCN [43] showed higher specificity than the proposed model by achieving 85.94%. The MS-AAGCN [40], CTR-GCN [43], and 2s-AGCN [39] showed higher precision than the proposed model by achieving 96.15%, 96.14%, and 95.61%, respectively. In the case of HCN, we excluded it from the comparison as it demonstrated an accuracy of less than 70% on this dataset.

Table 5 shows the results for the balanced group. The proposed model achieved 90.59% accuracy, 91.51% sensitivity, 89.06% specificity, and 93.27% precision. Similar to the results for the all-subject group, the accuracy and sensitivity of the proposed model were the highest among the models. The CTR-GCN [43] showed the second highest accuracy with 89.41%. DSTA-NET [42] achieved the second highest sensitivity of 90.57%. The specificity and precision of the proposed model were not the highest among the models. The CTR-GCN [43] and LSTM [20] showed higher specificity than the proposed model by achieving 92.19% and 90.63%, respectively. The CTR-GCN [43] and LSTM [20] also showed higher precision than the proposed model by achieving 94.90% and 93.33%, respectively.

We conducted an additional experiment to verify the effectiveness of using all of the gait parameters, joint angles, and skeleton data together. The encoding layers for the unused input data types were deactivated during the training, so the layers for only the used input data type affected the training. For example, the GCN layer was deactivated when the joint angles and the gait parameters were input to the model and the skeleton data were not used. The data of the balanced group were used for the evaluation, and the results are shown in Table 6. When a single type of input was fed to the model, using the skeleton data showed the best performance with 85.88% accuracy, 85.85% sensitivity, 85.94% specificity, and 91.00% precision. Using the gait parameters showed the second highest accuracy and sensitivity, with 79.41% and 80.19%, respectively. The results of using the joint angles showed the lowest accuracy and sensitivity of 72.35% and 64.15%, respectively. However, they showed higher specificity and precision than the results of using the gait parameters. When two types of input were fed to the model, the performance was improved compared with the results using the single type of input. Using the gait parameters and the skeleton data together showed the highest accuracy of 88.24%, which was the same as using the joint angles and the skeleton data together. Using the gait parameters and joint angles showed the lowest accuracy of 84.12%, which was 1.76% lower than the accuracy of using only the skeleton data. The highest sensitivity of 92.45% was achieved when using the joint angles and the skeleton data. The highest specificity and precision of 85.94% and 91.35%, respectively, were achieved when using the gait parameters and the skeleton data. When all types of input data were fed to the model, the highest accuracy, specificity, and precision were achieved compared to the results of using one or two types of input data. The sensitivity was lower than the results of using the joint angles and skeleton data.

## 4. Discussion

### 4.1. Principal Findings

We have uncovered several principal findings in the evaluation of our proposed model for skeleton-based pathological gait recognition through our experiments. We conducted assessments on both the simulated pathological gait dataset and the vestibular disorder gait dataset, each characterized by distinct gait abnormalities and subject configurations. This dual evaluation approach was crucial to assess the model’s robustness and versatility. The following findings promise to be a valuable contribution to the field of skeleton-based pathological gait recognition.

#### 4.1.1. Effectiveness of Integration of Gait Parameters, Joint Angles, and Skeleton Data

This study first tried to use all of the gait parameters, joint angles, and skeleton data together for pathological gait recognition. GCN, RNN, and ANN layers were used to effectively encode the skeleton sequences, joint angle sequences, and gait parameters, respectively. The model showed the best stable performance among the state-of-the-art models for skeleton-based action recognition on the different datasets. The skeleton data contain all information, so the maximum performance could theoretically be achieved by using the skeleton data alone. However, it is not easy for a model to understand the characteristics of pathological gaits using sequential skeleton data since the data are large and complicated. The gait features can be key to making a model better understand the skeleton data and to improve the performance. The gait parameters and joint angles are manually extracted features whose performances have been verified through various studies. If they are input to the model together with the skeleton data, they can help the model understand the skeleton data in a more effective way since they are the essence of human knowledge for pathological gait recognition.

#### 4.1.2. Performance Variation of Machine Learning Algorithms Based on Input Data Type

The machine learning algorithms were powerful when interpreting the gait parameters compared to the joint angles and skeleton data. Based on the results shown in Table 2, the average accuracies of the results of all machine learning algorithms for the gait parameters, joint angles, and skeleton data inputs were 82.33%, 71.86%, and 63.91%, respectively. The larger the volume of information was, the lower the performance of a machine learning-based classifier was. If the raw data were compressed to the features while preserving the important factors, the machine learning algorithms could better understand the distinguishable characteristics of the data and achieve better classification performance. However, there might be a loss of important factors when extracting features. Although the extracted features were powerful for the machine learning algorithms, the performance was lower than the results of feeding the raw skeleton sequences to the neural network-based classifiers.

#### 4.1.3. Differential Effects of Joint Angles and Gait Parameters Depending on Dataset

The joint angles were more effective than the gait parameters for the simulated pathological gait dataset, as shown in Table 2. However, contradictory results were obtained for the vestibular disorder gait dataset, as shown in Table 6. The effectiveness of the gait parameters and the joint angles depends on the pathological gait type to be recognized. The joint angle sequences could be more effective for pathological gaits related to motor functionalities, such as joint bending, muscle compensation, and postural balancing. Gait parameters might be more effective for pathological gaits, such as Parkinson’s disease and vestibular disorder gaits, which are related to sensory or cognitive functions and show irregular and unstable motions. Therefore, it is reasonable to use the gait parameters and joint angles together for the recognition of various gait abnormalities.

### 4.2. Comparison with Prior Work

The performances of existing studies for skeleton-based pathological gait recognition are as follows. Li et al. [3] classified normal controls, patients with hemiplegia, and patients with Parkinson’s disease with 79.0% accuracy using a k-NN classifier. They achieved high accuracy even in noisy and low-resolution data without calibration or synchronization requirements. Dranca et al. [4] recognized the stages of Parkinson’s disease with 93.40% accuracy using a Bayesian network with correlation-based feature selection. The key features for classification included left arm movement, trunk position during slightly displaced walking, and left shin angle for straight walking. An even higher accuracy of 96.23% was attained by focusing solely on features extracted from slightly displaced and spin walking steps. Seifallahi et al. [11] detected Alzheimer’s disease with an accuracy of 92.31%, sensitivity of 96.33%, precision of 88.62%, and specificity of 90.81% using an SVM with a Gaussian kernel. Guo et al. [17] classified normal, in-toeing, out-toeing, drop-foot, pronation, and supination gaits with 90.75% accuracy using bidirectional LSTM. They integrated this algorithm into a mobile robot system with potential applications in assisting elderly or neurologically impaired patients at home to reduce fall risks and improve their quality of life. Chakraborty et al. [23] recognized cerebral palsy gaits with 98.59% accuracy using an SVM with a radial basis function kernel. According to the ReliefF feature ranking algorithm, the walking ratio was identified as the highest-ranked feature among classical gait features, and its inclusion in the classification process substantially enhanced the performance of all classifiers. Chakraborty et al. [24] recognized equinus foot deformity gaits with 88.3% accuracy using a multiple adaptive regression splines model. To enhance accuracy, they created feature vectors using six joint angles, encompassing hip, knee, and ankle angles on both sides, and integrated these vectors over multiple time instances.

Our proposed model distinguishes itself from existing research in the field of skeleton-based pathological gait recognition through the introduction of a novel methodology that adeptly integrates a diverse range of input data. In terms of our model’s classification performance, it has exhibited exceptional proficiency in categorizing a broad spectrum of pathological gait types, encompassing normal, antalgic, steppage, lurching, stiff-legged, and Trendelenburg gaits, achieving an impressive accuracy rate of 99.03%. Furthermore, our model has demonstrated robust capabilities in discriminating vestibular disorder-related gaits, achieving accuracies of 91.03% in the all-subject group and 90.59% in the balanced group, confirming its applicability to real-world scenarios involving actual patients. However, it is essential to exercise caution when attempting to directly compare classification accuracies with previous studies within this context. This caution is warranted due to significant disparities in the pathological gait categories considered, the depth camera utilized for data acquisition, and the methodologies employed for pose estimation. Therefore, while our model’s achieved accuracy is undeniably impressive within the specialized scope of our study, it is advisable to avoid making direct comparisons with earlier research, given the substantial divergence in pathological gait typologies and dataset characteristics.

We have been steadily studying skeleton-based pathological gait recognition. In our first study [18], we improved the performance of pathological gait recognition by applying an RNN autoencoder to extract features from raw skeleton sequences. The automatically extracted features were more effective than the raw skeleton sequences. However, it took a long time for training since the RNN autoencoder and classification model were trained separately. The walking gait dataset [48] used in the research was composed of simple abnormal gait patterns created by padding a sole or attaching a weight, which were relatively easy to classify. Furthermore, the skeleton data were collected on a treadmill, so the subjects might not generate a natural gait pattern. For these reasons, we needed to collect complicated pathological gait data using our Kinect system without a treadmill. Therefore, in our second study [20], we collected complicated pathological gait patterns, i.e., antalgic, steppage, lurching, stiff-legged, and Trendelenburg gaits. We also proposed a GRU-based end-to-end model and applied a joint selection strategy to increase the performance. In our third study [49], we added a foot pressure sensor to our Kinect system. The performance of pathological gait recognition was further improved by using foot pressure and skeleton data together. However, the multimodal system was complicated, and the foot pressure sensor was costly, so it was not suitable for practical use. Therefore, we studied a method for maximizing pathological gait recognition performance using a single Kinect sensor and eventually arrived at the hybrid model proposed in this study.

### 4.3. Limitations and Future Works

This study had several limitations. First, the proposed hybrid model was validated on the limited types of pathological gaits. In the future, we should collect abnormal gait data with diverse diseases, such as Parkinson’s disease, autism spectrum disorder, stroke, Alzheimer’s disease, sarcopenia, and functional gait disorders, and validate the proposed model on those data to verify the application validity on other pathological gaits. Second, we focused on skeleton-based pathological gait recognition, so we only used skeleton-induced gait parameters, whereas there are many other clinical parameters, such as body composition analysis, hemanalysis, video head impulse test, videonystagmography, Montreal cognitive assessment, mini-mental state exam, time up and go test, or the Tinetti test. Therefore, in the future, we plan to collect those clinical parameters and apply them to the proposed model to improve the performance of pathological gait recognition. Third, we just concatenated the encoded features and fed them to the classification layer. In the future, we aim to further enhance our method by implementing adaptive feature selection or weighting techniques. This will involve the selection or assignment of weights to features and matching scores that demonstrate low correlation and high discrimination. Fourth, the ST-GCN, LSTM, and ANN are effective neural network architectures to encode the skeleton data, joint angle sequences, and gait parameters, respectively, but might not be the best neural network architectures to encode each of them. The proposed model can be further improved by replacing the encoding layers with advanced algorithms optimized for each input data type. In the future, we will continue to modify the current neural network architectures with the latest algorithm and reflect on it in future works.

## 5. Conclusions

The proposed hybrid deep neural network, which effectively used gait parameters, joint angles, and skeleton data, improved the performance of pathological gait recognition on two different datasets. The proposed model not only classified the diverse pathological gaits (simulated) but also recognized the gait abnormalities of real patients with a vestibular disorder. The fusion of the different inputs had a positive synergy on pathological gait recognition by integrating the features based on human knowledge and those automatically extracted by artificial intelligence. The framework can provide inspiration for the development of skeleton-based pathological gait recognition models. Furthermore, it can be flexibly modified by replacing the encoding layers or adding clinical information, which can further improve the performance. In the future, we will collect skeleton datasets for patients with other diseases, such as Parkinson’s disease and sarcopenia, and evaluate the performance when classifying different diseases to verify the practical use and expansion of the application.

## Figures and Tables

**Table 1:** The detailed configuration of the hybrid deep neural network architecture.

Type	Layer	Configuration
GCN Encoding Layers	ST-GCN	In_channels = 3, out_channels = 64, kernel_size = (9,3)
	ST-GCN	In_channels = 64, out_channels = 64, kernel_size = (9,3)
	ST-GCN	In_channels = 64, out_channels = 64, kernel_size = (9,3)
	ST-GCN	In_channels = 64, out_channels = 64, kernel_size = (9,3)
	ST-GCN	In_channels = 64, out_channels = 128, kernel_size = (9,3)
	ST-GCN	In_channels = 128, out_channels = 128, kernel_size = (9,3)
	ST-GCN	In_channels = 128, out_channels = 128, kernel_size = (9,3)
	ST-GCN	In_channels = 128, out_channels = 256, kernel_size = (9,3)
	ST-GCN	In_channels = 256, out_channels = 256, kernel_size = (9,3)
	ST-GCN	In_channels = 256, out_channels = 256, kernel_size = (9,3)
	Average Pooling	/
	2D Convolution	In_channels = 256, out_channels = 64, kernel_size = 1
	Flatten	/
	ReLU Activation	Hidden unit = 64
RNN Encoding Layers	LSTM	Hidden unit = 128, return_sequences = True
	LSTM	Hidden unit = 128, return_sequences = True
	LSTM	Hidden unit = 128, return_sequences = True
	LSTM	Hidden unit = 128, return_sequences = False
	Batch Normalization	/
	Fully Connected Layer	Hidden unit = 16
	ReLU Activation	/
ANN Encoding Layers	Fully Connected Layer	Hidden unit = 16
	ReLU Activation	/
Classification Layers	Batch Normalization	/
	Dropout	Ratio = 0.5
	Fully Connected Layer	Hidden unit = the number of classes
	Softmax	/

**Table 2:** Accuracy of single input models on the simulated pathological gait dataset.

Model	Input Data		
	Gait Parameters (%)	Joint Angles (%)	Skeleton (%)
AdaBoost	76.20	56.70	55.24
decision tree	74.54	57.05	55.28
Gaussian Naïve Bayes	83.61	79.86	58.19
random forest	87.18	82.26	67.67
k-NN	83.98	76.46	74.44
SVM	88.47	78.82	72.64
MLP	**90.49**	79.97	86.11
GRU [20]	/	92.92	95.83
LSTM [20]	/	**93.30**	95.20
ST-GCN [36]	/	/	**96.94**

**Table 3:** Accuracy of the state-of-the-art models and the proposed model on the simulated pathological gait dataset.

Model	Accuracy (%)
LSTM [20]	95.20
GRU [20]	95.83
ST-GCN [36]	96.94
HCN [37]	96.07
Decouple GCN [38]	96.25
2s-AGCN [39]	98.06
MS-AAGCN [40]	97.78
PB-GCN [41]	97.53
DSTA-NET [42]	97.91
CTR-GCN [43]	98.75
Proposed	**99.03**

**Table 4:** Performance on the all-subject group of the vestibular disorder gait dataset.

Model	All-Subject Group							
	Accuracy (%)	Sensitivity (%)	Specificity (%)	Precision (%)	TP	FP	TN	FN
LSTM [20]	84.94	87.50	75.00	93.13	217	16	48	31
GRU [20]	85.26	87.10	78.13	93.91	216	14	50	32
ST-GCN [36]	85.90	88.31	76.56	93.59	219	15	49	29
Decouple GCN [38]	86.22	87.10	82.81	95.15	216	11	53	32
2s-AGCN [39]	87.18	87.90	84.38	95.61	218	10	54	30
MS-AAGCN [40]	89.74	90.73	**85.94**	**96.15**	225	9	55	23
PB-GCN [41]	85.26	89.52	68.75	91.74	222	20	44	26
DSTA-NET [42]	86.86	91.94	67.19	91.57	228	21	43	20
CTR-GCN [43]	89.42	90.32	**85.94**	96.14	224	9	55	24
Proposed	**91.03**	**93.15**	82.81	95.45	231	11	53	17

**Table 5:** Performance on the balanced group of the vestibular disorder gait dataset.

Model	Balanced Group							
	Accuracy (%)	Sensitivity (%)	Specificity (%)	Precision (%)	TP	FP	TN	FN
LSTM [20]	83.53	79.25	90.63	93.33	84	6	58	22
GRU [20]	84.12	83.96	84.38	89.90	89	10	54	17
ST-GCN [36]	85.88	85.85	85.94	91.00	91	9	55	15
Decouple GCN [38]	86.47	86.79	85.94	91.09	92	9	55	14
2s-AGCN [39]	87.06	87.74	85.94	91.18	93	9	55	13
MS-AAGCN [40]	88.24	88.68	87.50	92.16	94	8	56	12
PB-GCN [41]	88.24	87.74	89.06	93.00	93	7	57	13
DSTA-NET [42]	85.88	90.57	78.13	87.27	96	14	50	10
CTR-GCN [43]	89.41	87.74	**92.19**	**94.90**	93	5	59	13
Proposed	**90.59**	**91.51**	89.06	93.27	97	7	57	9

**Table 6:** Performance of the proposed model on the balanced group of the vestibular disorder gait dataset when changing the combination of the inputs.

Number of Input Types	Input Type	Accuracy (%)	Sensitivity (%)	Specificity (%)	Precision (%)
1	Gait parameters	79.41	80.19	78.13	85.86
	Joint angles	72.35	64.15	85.94	88.31
	Skeleton	85.88	85.85	85.94	91.00
2	Gait parameters, joint angles	84.12	84.91	82.81	89.11
	Gait parameters, skeleton	88.24	89.62	85.94	91.35
	Joint angles, skeleton	88.24	**92.45**	81.25	89.09
3	Gait parameters, joint angles, skeleton	**90.59**	91.51	**89.06**	**93.27**

## Data Availability

Not applicable.
